# A pre-trained foundation model framework for multiplanar MRI classification of extramural vascular invasion and mesorectal fascia invasion in rectal cancer

**DOI:** 10.1186/s13244-026-02296-3

**Published:** 2026-05-22

**Authors:** Yumeng Zhang, Shruti Atul Mali, Danial Khan, Sina Amirrajab, Eduardo Ibor-Crespo, Ana Jimenez-Pastor, Gloria Ribas, Silvia Flor-Arnal, Marta Zerunian, Christophe Aubé, Luis Martí-Bonmatí, Zohaib Salahuddin, Philippe Lambin

**Affiliations:** 1 The D-Lab, Department of Precision Medicine, GROW - Research Institute for Oncology and Reproduction, Maastricht University, Maastricht, The Netherlands; 2 Research & Frontiers in AI Department, Quantitative Imaging Biomarkers in Medicine, Quibim SL, Valencia, Spain; 3 Biomedical Imaging Research Group, La Fe Health Research Institute, Valencia, Spain; 4 Radiology Unit, Department of Surgical and Medical Sciences and Translational Medicine, Sapienza University of Rome, Sant’Andrea Hospital, Rome, Italy; 5 Laboratoire HIFIH, Université d’Angers, SFR ICAT 4208, Angers, France; 6 Department of Radiology, CHU Angers, Angers, France; 7 Medical Imaging Department, La Fe University and Polytechnic Hospital, Valencia, Spain; 8 Department of Radiology and Nuclear Medicine, GROW - Research Institute for Oncology and Reproduction, Maastricht University Medical Center+, Maastricht, The Netherlands

**Keywords:** Rectal neoplasms, Magnetic resonance imaging, Extramural vascular invasion, Mesorectal fascia, Deep learning

## Abstract

**Objectives:**

Accurate MRI-based identification of extramural vascular invasion (EVI) and mesorectal fascia invasion (MFI) is crucial for risk-stratified rectal cancer treatment. However, subjective visual assessment and inter-institutional variability limit diagnostic consistency. This study developed and evaluated a multi-center, foundation model-driven framework that automatically classifies EVI and MFI on axial and sagittal MRI.

**Materials and methods:**

A total of 331 pre-treatment rectal cancer T2-weighted MRI scans from three European hospitals were retrospectively recruited. A self-supervised frequency domain harmonization strategy was applied to reduce scanner variability. Three classifiers, SeResNet, the universal biomedical pretrained model (UMedPT) with a multilayer perceptron head, and a logistic-regression variant using frozen UMedPT features (UMedPT_LR), were trained (*n* = 265) and tested (*n* = 66). Gradient-weighted class activation mapping (Grad-CAM) visualized model predictions.

**Results:**

UMedPT_LR achieved the best EVI performance with multiplanar fusion (AUC = 0.82, test set). For MFI, UMedPT trained on axial harmonized images yielded the highest performance (AUC = 0.77). Both tasks outperformed the CHAIMELEON 2024 benchmark (EVI: 0.82 vs 0.74; MFI: 0.77 vs 0.75). Harmonization enhanced MFI classification, and multiplanar fusion further boosted EVI performance. Grad-CAM confirmed biologically plausible attention on peritumoral regions (EVI) and mesorectal fascia margins (MFI).

**Conclusion:**

The proposed foundation model-driven framework, leveraging frequency domain harmonization and multiplanar fusion, achieves state-of-the-art performance for automated EVI and MFI classification on MRI, demonstrating strong generalizability across multiple centers.

**Critical relevance statement:**

Addressing inter-center inconsistencies in rectal cancer MRI, a multiplanar foundation model with cross-scanner harmonization significantly improves the detection of EVI and MFI, potentially standardizing staging and guiding therapy.

**Key Points:**

- Among the first studies to investigate automated classification of both EVI and MFI using axial and sagittal T2-weighted MRI.
- Foundation model-derived features outperform conventional convolutional neural networks (CNNs) for EVI and MFI classification.
- Frequency domain harmonization and multiplanar fusion selectively enhance diagnostic performance.
- Automated prediction of EVI and MFI may support more consistent staging and clinical decision-making across institutions.

**Graphical Abstract:**

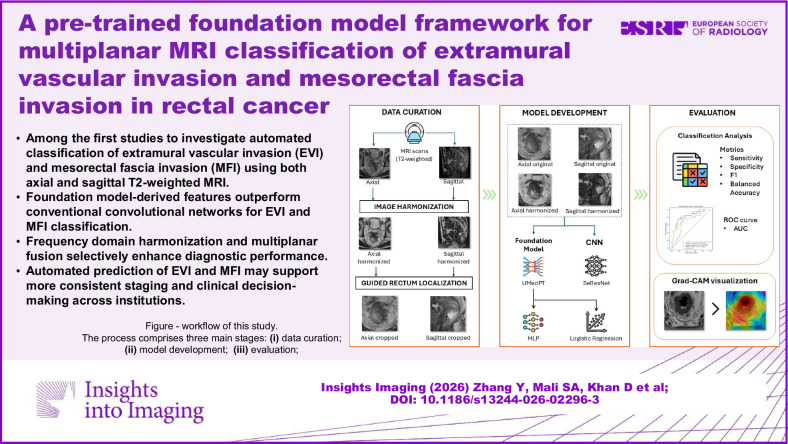

## Introduction

Rectal cancer accounts for approximately one-third of colorectal cancer cases and remains a major cause of cancer-related mortality worldwide [1–3]. Accurate staging is essential for tailoring treatment strategies and improving patient outcomes [4]. Among key prognostic markers, extramural vascular invasion (EVI) and mesorectal fascia invasion (MFI) play critical roles in guiding therapeutic decisions. EVI refers to the extension of tumor cells beyond the muscularis propria into perirectal vessels and is strongly associated with local recurrence, distant metastasis, and poor overall survival. Its presence can influence management even in node-negative patients, supporting the recommendation of neoadjuvant chemoradiotherapy (nCRT) to reduce recurrence risk [5]. MFI describes tumor involvement of the mesorectal fascia, the connective tissue envelope surrounding the rectum. Identification of MFI indicates a high risk of an involved circumferential resection margin (CRM), prompting guidelines to recommend nCRT to downstage the tumor and enhance the likelihood of a clear CRM during surgery [6].

MRI provides direct visualization of EVI and MFI and is considered the gold standard for locoregional staging of rectal cancer [7]. On T2-weighted MRI, EVI appears as irregular extensions beyond the muscularis propria into perirectal vessels [8]. While axial images best depict these vascular protrusions [9], sagittal images provide complementary information on the longitudinal extent of vascular involvement [10]. By contrast, MFI is characterized by loss of the normally sharp mesorectal fascia, reflecting tumor extension toward or into this boundary [11]. Although sagittal images are less precise for defining fascial margins, they remain valuable for assessing craniocaudal tumor spread and its relationship to the anal canal and pelvic floor [12]. Collectively, these perspectives underscore the relevance of multiplanar MRI assessment for accurate evaluation of both EVI and MFI.

While experienced radiologists are able to identify these features on T2-weighted MRI, interpretation is subjective, varies between institutions, and becomes particularly variable in borderline cases. These limitations highlight the need for artificial intelligence (AI)-based approaches that provide objective and reproducible assessments, thereby supporting consistent staging decisions [13].

Radiomics and deep learning have emerged as promising strategies for extracting high-dimensional imaging features imperceptible to the human eye [14–16]. These methods have demonstrated clinical utility in predicting treatment response, nodal metastasis, and survival in rectal cancer patients [17]. However, standard deep learning models are highly sensitive to inter-institutional variability in scanner type and acquisition protocols [18, 19], particularly in multi-center studies [20]. To address this challenge, harmonization methods such as ComBat [21] or CycleGAN [22] have been proposed, whereas more recent frequency domain and self-supervised approaches aim to learn modality-invariant representations with minimal supervision [23].

Recently, foundation models trained on large and heterogeneous medical image datasets have emerged as a promising approach to mitigate these limitations by enabling generalizable and transferable feature learning [24]. For instance, RETFound [25] for retinal imaging and BEPH [26] for histopathology demonstrate how large-scale pretraining can yield transferable features. However, these models remain limited to modality-specific applications. The Universal Biomedical Pretrained Model (UMedPT [27]) is a cross-domain foundation model designed to support diagnostic tasks across diverse biomedical imaging modalities. Its potential has not yet been explored for rectal MRI classification tasks.

In this study, we propose a foundation model-based framework for automated classification of EVI and MFI in rectal cancer using axial and sagittal T2-weighted MRI from multiple vendors and centers. Our approach leverages the UMedPT foundation model alongside a logistic regression classifier applied to deep features extracted from UMedPT (UMedPT_LR). To mitigate inter-institutional variability, we incorporate a self-supervised frequency domain harmonization strategy, and to enhance interpretability, we apply Gradient-weighted class activation mapping (Grad-CAM) to visualize the spatial rationale of model predictions.

## Methods

**Fig. 1:**
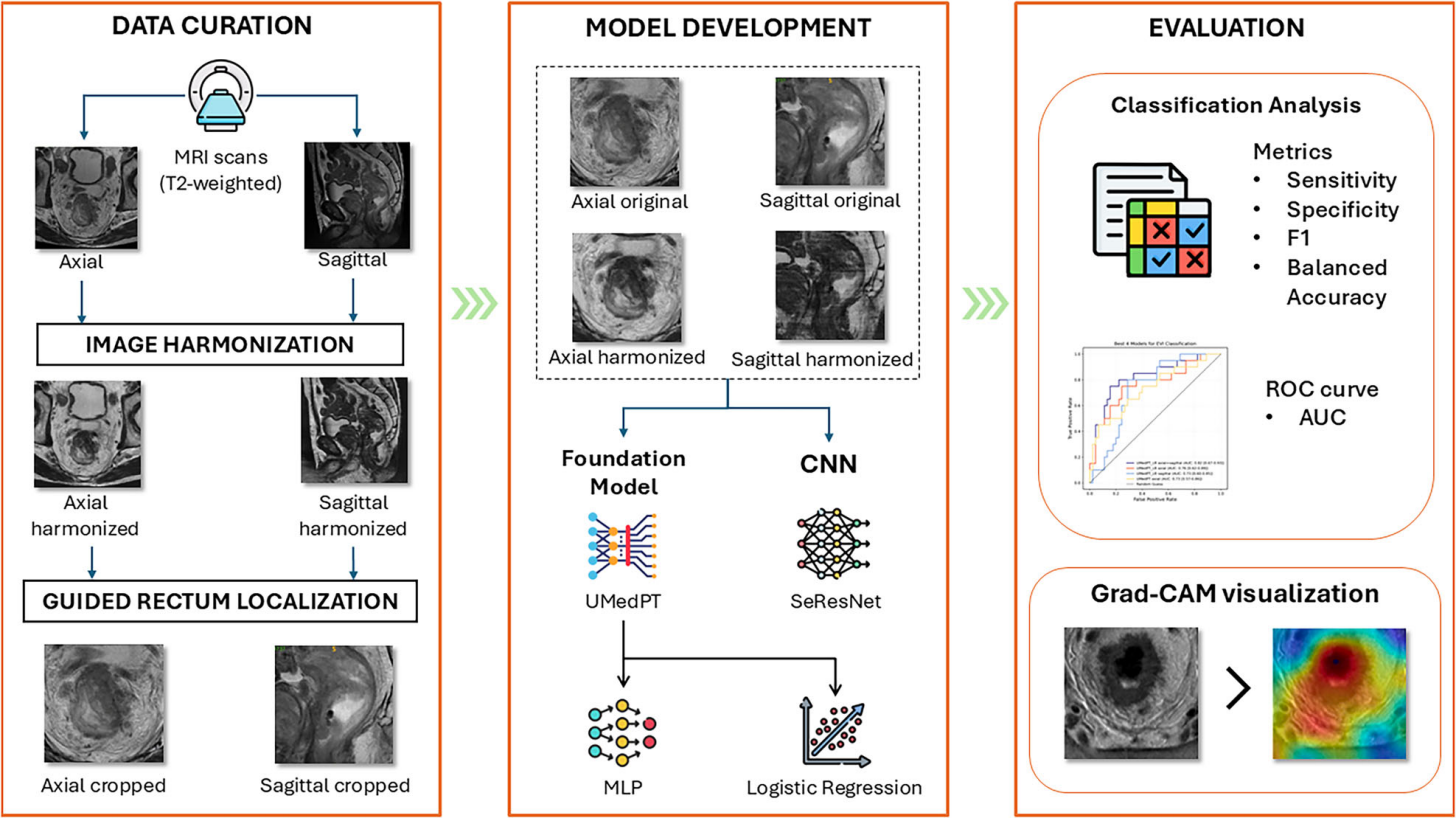
Workflow of this study. The process comprises three main stages: (i) data curation, including acquisition of axial and sagittal T2-weighted MRI images, image harmonization to reduce inter-scanner variability, and guided localization of the rectum; (ii) model development, where both the original and harmonized images were processed using a foundation model (UMedPT) and a convolutional neural network (SeResNet), followed by classification using either a multilayer perceptron (MLP) or logistic regression; and (iii) evaluation, involving classification performance analysis with multiple metrics (sensitivity, specificity, F1 score, and balanced accuracy), ROC curves with AUC computation, and model interpretability assessment via Grad-CAM visualization

This study followed a structured workflow encompassing data curation, model development, and evaluation, with the overall study design and analytical pipeline illustrated in Fig. 1.

### Data curation

#### Dataset

This retrospective study utilized data from the European CHAIMELEON [28] project, a large-scale initiative aimed at developing AI algorithms for cancer diagnosis across multiple tumor types, including lung, colon, breast, prostate, and rectum cancers. All imaging and clinical data were processed within the CHAIMELEON platform, which ensured secure handling and compliance with privacy regulations. In this study, we included 331 rectal cancer patients from three hospitals—La Fe University and Polytechnic Hospital (La Fe, *n* = 194), Sant’Andrea University Hospital, Sapienza University of Rome (ULS, *n* = 113), and Centre Hospitalier Universitaire d’Angers (CHU Angers, *n* = 24)—utilizing MRI scanners from mainly two manufacturers—GE (*n* = 221) and Siemens (*n* = 106).

Patient inclusion criteria were: (1) biopsy-confirmed rectal adenocarcinoma; (2) age ≥ 18 years (diagnosis year ≥ 2014); (3) No distant metastasis at baseline (M0); and (4) availability of pre-treatment pelvic MR exam with axial and sagittal T2-weighted images. (5) Patients were followed ≥ 12 months from the first treatment, unless death, recurrence, or progression occurred earlier. This follow-up criterion was applied to ensure data completeness and adequate clinical documentation; study endpoints were diagnostic rather than longitudinal. Exclusion criteria were absence of baseline pelvic MR (*n* = 12) or incomplete sequences (*n* = 4).

**Fig. 2:**
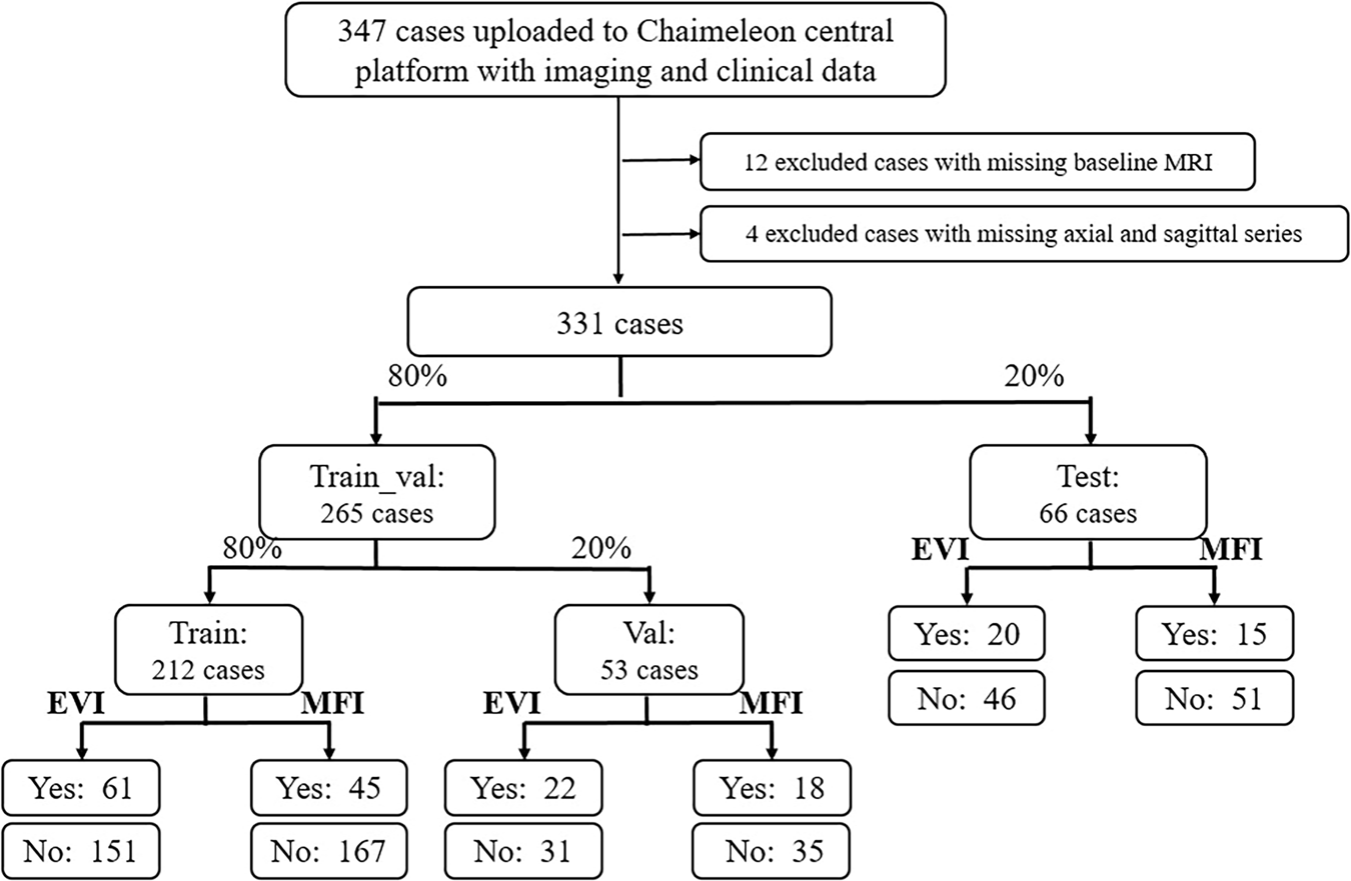
Flowchart of data inclusion, exclusion, and dataset stratification for model development and evaluation

As illustrated in Fig. 2, the data partitions followed the predefined splits provided by the CHAIMELEON challenge organizers. The original challenge cohorts consisted of a training set (*n* = 231), a validation set (*n* = 34), and an independent test set (*n* = 66). In this study, the original training and validation cohorts were combined into a single train_val set (*n* = 265). Within this train_val cohort, 80% of the cases (*n* = 212) were used for model training and the remaining 20% (*n* = 53) for internal validation. The test set (*n* = 66) remained unchanged from the original challenge split to enable direct comparison with other studies using the same benchmark. Ground truth labels for EVI and MFI were derived from pre-treatment MRI structured radiology reports. Pathological CRM status (involved/uninvolved) was extracted from histopathology reports and used as an ancillary clinicopathological variable. All cases were evaluated by two radiologists. The first radiologist, a specialist, provided the original clinical report for the clinical practice. A second radiologist conducted a dedicated review within the framework of the present study.

#### Image preprocessing and rectum localization

Data were converted from DICOM to NIfTI and processed with a structured automatic pipeline. Pelvic anatomy was segmented using TotalSegmentator [29]. The colon mask served as the primary cue for rectal localization, with adjacent structures (e.g., bladder) used as auxiliary references. We computed the rectal centroid within the colon mask and applied a fixed-size center-crop patch to both axial and sagittal planes, ensuring consistent rectal coverage and alignment across patients. All series were resampled to a uniform voxel spacing of 0.39 × 0.39 × 3.3 mm to standardize resolution across scans. To reduce inter-patient intensity variation, voxel values were clipped to the 2.5–97.5th percentiles and subsequently *Z*-score normalized.

#### Frequency domain harmonization

To address inter-institutional variability in multi-center T2w MR dataset, we employed a frequency domain harmonization pipeline developed by QUIBIM as a part of the CHAIMELEON project. A curated reference set defined the target contrast distribution, synthetic variants were generated by Fourier-domain perturbations, and a self-supervised UNet-based autoencoder learned to reconstruct standardized images from perturbed inputs. The resulting outputs were used alongside originals in downstream classification. Complete specifications and training schedule are present in Appendix A1.

### Model development

#### SeResNet

We employed a modified version of Squeeze and Excitation Residual Network (SeResNet)-34 with an anisotropic stem (3 × 3 × 1) and early (2,2,1) strides to respect through-plane resolution in pelvic MRI. The network ends with global average pooling and a sigmoid output for binary EVI/MFI classification. Full layer configuration and training settings are in Appendix A2.

#### UMedPT

**Fig. 3:**
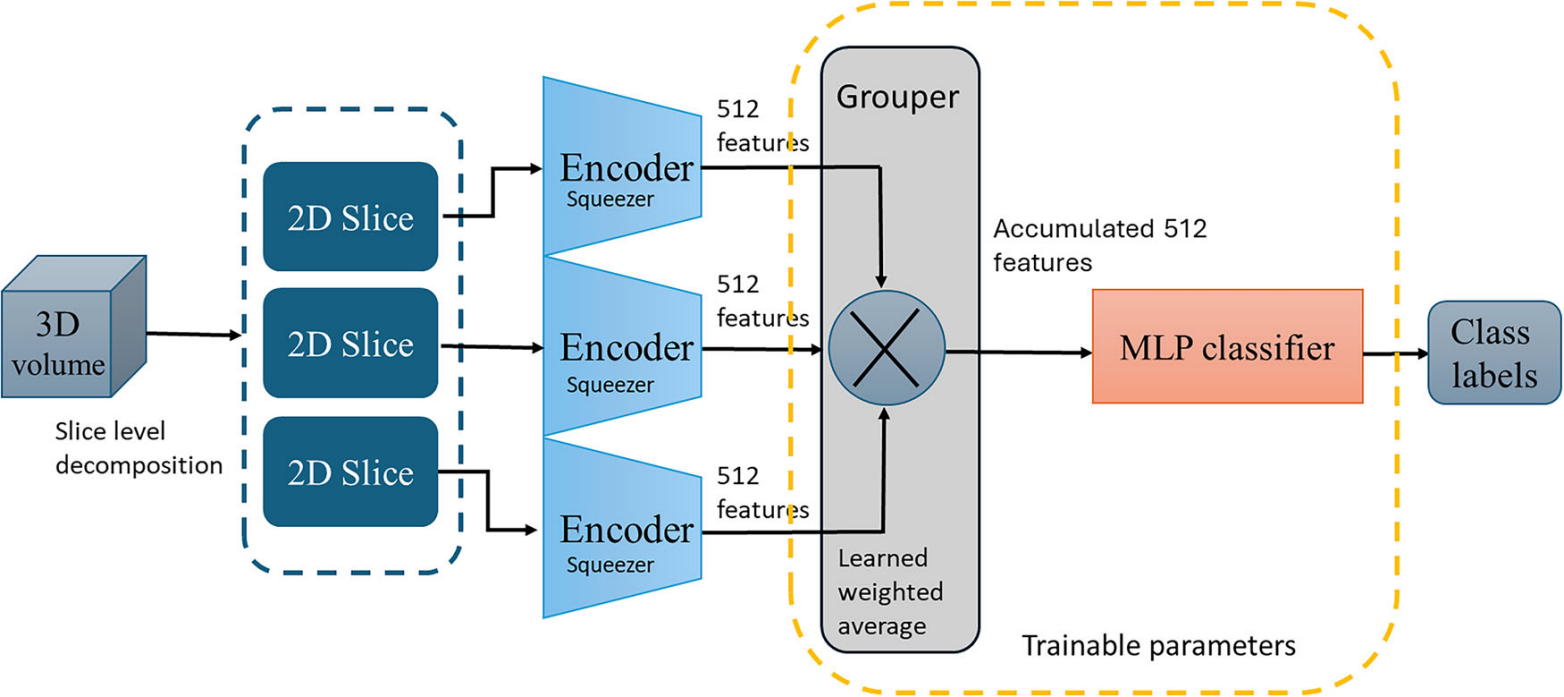
UMedPT architecture for rectal cancer stage classification using multi-slice MRI. Each 3D MRI volume is decomposed into 2D slices, which are individually encoded and compressed via shared encoders and squeezer modules pretrained in UMedPT. The extracted 512-dimensional features from all slices are aggregated using a learned weighting strategy in the Grouper module. The accumulated representation is then passed to an MLP classifier to predict the presence or absence of EVI and MFI. During fine-tuning, only the grouper and classifier parameters are updated, while the backbone encoder remains frozen

UMedPT [27] is a foundation model designed to learn transferable representations across diverse biomedical imaging modalities and tasks. In this study, we adapt UMedPT for the classification of EVI and MFI from T2-weighted MRI pelvic images. As illustrated in Fig. 3, the input 3D volume was first decomposed into a stack of 2D slices. Each slice is processed independently through a shared encoder-squeezer module pretrained within the UMedPT framework. The encoder, based on the Swin Transformer architecture, extracts high-level modality-agnostic features and produces a hierarchical representation with 288 tokens, each compressed into a 512-dimensional feature vector by the squeezer. These features are subsequently aggregated by the grouper into a single 512-dimensional representation for classification. This is followed by a Multilayer perceptron (MLP) classifier, consisting of two fully connected layers with ReLU activations and dropout, which outputs binary predictions for EVI or MFI.

During fine-tuning, only the grouper and classifier layers were optimized, while the encoder and squeezer components were frozen to preserve pretrained feature representations. Input images were duplicated across three channels to conform to the encoder’s requirements. This architecture enables efficient transfer learning and robust volume-level classification, while minimizing the number of trainable parameters required for domain adaptation.

#### UMedPT_LR and multiplanar fusion

UMedPT_LR replaces the MLP head with a logistic regression classifier applied to the 512-dimensional UMedPT grouper features. Feature dimensionality was reduced using principal component analysis (PCA) before fitting the logistic-regression model. This design lowers computational cost and enhances interpretability through component-level coefficients.

In the multiplanar fusion setting, we concatenate the 512-dimensional UMedPT grouper features from the axial and sagittal models into a 1024-dimensional vector. This fused representation then undergoes the same pipeline as UMedPT_LR.

#### Training strategy

Data augmentation (MONAI [30]) comprised random affine transforms, intensity adjustments, Gaussian noise and smoothing, and MRI-specific artifacts. Detailed settings are provided in Appendix A3. All images were rescaled to the 2.5th–97.5th intensity percentile range. A patch size of 192 × 192 × 36 was employed to capture the full lesion context while minimizing interference from surrounding anatomy.

SeResNet was optimized using α-balanced focal loss and trained for 200 epochs using SGD with a learning rate of 1 × 10⁻³. A warm-up of 10 epochs and ReduceLROnPlateau scheduling were used. UMedPT used a weighted binary cross-entropy loss and AdamW optimizer with a learning rate of 5 × 10⁻⁴. Cosine annealing was used for learning rate decay. Both models were trained with a batch size of 8 using half-precision floating-point arithmetic.

### Evaluation

#### Evaluation metrics

Model performance was evaluated using the area under the curve (AUC) as the primary metric, alongside balanced accuracy, sensitivity, specificity, and F1 score to account for class imbalance. Bootstrap resampling (1000 iterations) was used to compute 95% confidence intervals (CI), ensuring robust and reliable estimates. Sensitivity and specificity were computed from binary predictions, obtained by thresholding the predicted probabilities at 0.5. In practical deployment, the operating threshold may be selected on the validation set, for example, by maximizing the Youden index or by choosing a target-sensitivity operating point depending on the clinical objective.

Sensitivity and specificity are defined as:

1 $${Sensitivity}=\frac{{TP}}{{TP}+{FN}}$$

2 $${Specificity}=\frac{{TN}}{{TN}\,+{FP}}$$

Balanced accuracy accounts for both sensitivity and specificity, offering a fairer evaluation when class distributions are skewed:

3 $${Balanced}\,a{ccuracy}=\frac{\left({Sensitivity}\,+\,{Specificity}\right)}{2}$$

The *F*1 score provides the harmonic mean of precision and recall:

4 $$F1=\frac{{TP}}{{TP}\,+\,\frac{1}{2}\left({FP}\,+\,{FN}\right)}$$

#### Model interpretability

To interpret the spatial rationale underlying model decisions, we applied Grad-CAM [31] to the trained UMedPT-based classifier. Specifically, for each axial original T2-weighted MRI scans, the input image was normalized and converted to RGB format. Grad-CAM heatmaps were generated by targeting the norm1 layer of the first SwinTransformerBlockV2 (the hierarchical transformer module within the UMedPT encoder) in the 7th sequential block, which corresponds to high-level contextual features. The resulting heatmaps were normalized and overlaid on the original MRI slices.

#### Reporting quality assessment

We evaluated methodological quality using the Radiomics Quality Score 2.0 (RQS 2.0) [32]. This framework provides standardized criteria to assess transparency, reproducibility, and clinical applicability in radiomics and AI-based imaging studies.

## Results

### Patient characteristics

The characteristics of the patient cohort are summarized in Table 1. The median age of the patients was 65 years (range: 28–89 years), and 60.7% (*n* = 201) were male. EVI was positive in 31.1% (*n* = 103) of cases, while MFI was positive in 23.6% (*n* = 78). Patients were recruited primarily from three hospitals: LaFe (58.6%, *n* = 194), ULS (34.1%, *n* = 113), and CHU Angers (7.3%, *n* = 24). MRI scans were predominantly obtained using GE scanners (66.8%, *n* = 221), followed by Siemens scanners (32.0%, *n* = 106), with a small minority from other manufacturers (1.2%, *n* = 4). The demographic, clinical, institutional, and scanner characteristics were comparable across the train_val, and test cohorts. No statistically significant differences were observed between the cohorts (all *p* > 0.05).

**Table 1:** Patient characteristics

Characteristics	All	Train_Val	Test	*p* value
Number of patients	331	265	66	
Age (median, range)	65 (28–89)	65 (29–89)	65 (28–87)	0.80
Gender (male)	201 (60.7%)	162 (61.1%)	39 (59.1%)	0.78
Type of rectal cancer				
EVI (+)	103 (31.1%)	83 (31.3%)	20 (30.3%)	1.00
MFI (+)	78 (23.6%)	63 (23.8%)	15 (22.7%)	1.00
Medical center				0.59
La Fe	194 (58.6%)	158 (59.6%)	36 (54.5%)	
ULS	113 (34.1%)	87 (32.8%)	26 (39.4%)	
CHU Angers	24 (7.3%)	20 (7.5%)	4 (6.1%)	
Manufacturer				0.10
GE	221 (66.8%)	183 (69.1%)	38 (57.6%)	
Siemens	106 (32.0%)	80 (30.2%)	26 (39.4%)	
Other	4 (1.2%)	2 (0.7%)	2 (3.0%)	

Values for age are median (range); all other values are counts with percentages in parentheses. *p* values were calculated using the Mann–Whitney U test (age), Fisher’s exact test (gender, EVI, MFI), and Chi-square tests (medical center, manufacturer)

*La Fe* La Fe University and Polytechnic Hospital, *ULS* Sant’Andrea University Hospital, Sapienza University of Rome, *CHU Angers* Center Hospitalier Universitaire d’Angers, *EVI* extramural vascular invasion, *MFI* mesorectal fascia invasion

^+^ Indicates presence of the feature

### Classification results

We evaluated SeResNet, UMedPT, and UMedPT_LR for classifying EVI and MFI on axial and sagittal T2-weighted MRI, with and without harmonization. All performance metrics reported below were obtained on the test set. Overall, our models consistently outperformed the CHAIMELEON challenge winner’s model [28], demonstrating superior generalizability across orientations and preprocessing conditions.

For EVI classification, UMedPT_LR achieved the strongest performance. On single-plane patches, it obtained an AUC of 0.76 (balanced accuracy 0.70) on axial images and 0.73 (balanced accuracy 0.68) on sagittal images, surpassing both SeResNet and UMedPT (Table 2). When axial and sagittal planes were fused, the UMedPT_LR model achieved the highest performance with an AUC of 0.82, sensitivity of 0.75, and F1 score of 0.73 (Table 3). As illustrated in the receiver operating characteristic (ROC) curves (Fig. 5, left), the fused model provided the best discrimination among all evaluated approaches and outperformed the CHAIMELEON challenge winner (AUC = 0.74). Notably, harmonization impaired EVI detection, with performance decreasing substantially across both orientations.

**Table 2:** Diagnostic performance of different models for classifying EVI on axial and sagittal T2-weighted MRI, before and after harmonization (test set)

Plane	Model	AUC (95% CI)	Sensitivity (95% CI)	Specificity (95% CI)	F1 (95% CI)	Balanced_acc (95% CI)
Axial original	SeResnet	0.57 (0.41–0.71)	0.70 (0.47–0.89)	0.44 (0.31–0.59)	0.48 (0.30–0.63)	0.57 (0.44–0.70)
	UMedPT	0.73 (0.57–0.86)	0.65 (0.43–0.85)	0.67 (0.53–0.81)	0.54 (0.35–0.70)	0.66 (0.53–0.78)
	UMedPT_LR	0.76 (0.62–0.89)	0.60 (0.35–0.82)	0.80 (0.68–0.91)	0.59 (0.36–0.74)	0.70 (0.58–0.82)
Axial harmonized	SeResnet	0.60 (0.45–0.75)	0.85 (0.67–1.0)	0.36 (0.22–0.50)	0.52 (0.36–0.65)	0.60 (0.49–0.70)
	UMedPT	0.65 (0.49–0.80)	0.60 (0.37–0.80)	0.64 (0.51–0.79)	0.50 (0.32–0.67)	0.62 (0.49–0.75)
	UMedPT_LR	0.64 (0.47–0.78)	0.45 (0.22–0.67)	0.71 (0.58–0.84)	0.43 (0.21–0.61)	0.58 (0.45–0.71)
Sagittal original	SeResnet	0.52 (0.35–0.68)	0.55 (0.31–0.75)	0.58 (0.43–0.72)	0.44 (0.25–0.59)	0.56 (0.42–0.69)
	UMedPT	0.67 (0.53–0.82)	0.50 (0.28–0.74)	0.71 (0.58–0.84)	0.47 (0.26–0.64)	0.61 (0.47–0.74)
	UMedPT_LR	0.73 (0.60–0.85)	0.60 (0.38–0.81)	0.76 (0.63–0.87)	0.56 (0.35–0.73)	0.68 (0.55–0.80)
Sagittal harmonized	SeResnet	0.63 (0.47–0.79)	0.70 (0.49–0.91)	0.53 (0.39–0.68)	0.51 (0.33–0.67)	0.62 (0.49–0.74)
	UMedPT	0.53 (0.36–0.69)	0.0 (0.0–0.0)	1.0 (1.0–1.0)	0.0 (0.0–0.0)	0.50 (0.50–0.50)
	UMedPT_LR	0.55 (0.38–0.72)	0.35 (0.15–0.57)	0.80 (0.69–0.91)	0.39 (0.18–0.57)	0.58 (0.45–0.69)

**Table 3:** Diagnostic performance of multiplanar fusion for UMedPT_LR model on EVI and MFI classification (test set)

Multiplanar fusion	AUC (EVI)	AUC (MFI)	Sensitivity (EVI)	Sensitivity (MFI)	F1 (EVI)	F1 (MFI)
Axial original + sagittal original	0.82 (0.69–0.93)	0.71 (0.55–0.84)	0.75 (0.48–0.89)	0.73 (0.41–0.90)	0.73 (0.50–0.83)	0.51 (0.27–0.67)
Axial harmonized + sagittal harmonized	0.58 (0.44–0.72)	0.75 (0.61–0.87)	0.35 (0.15–0.57)	0.07 (0.0–0.23)	0.37 (0.17–0.55)	0.13 (0.0–0.38)

**Fig. 4:**
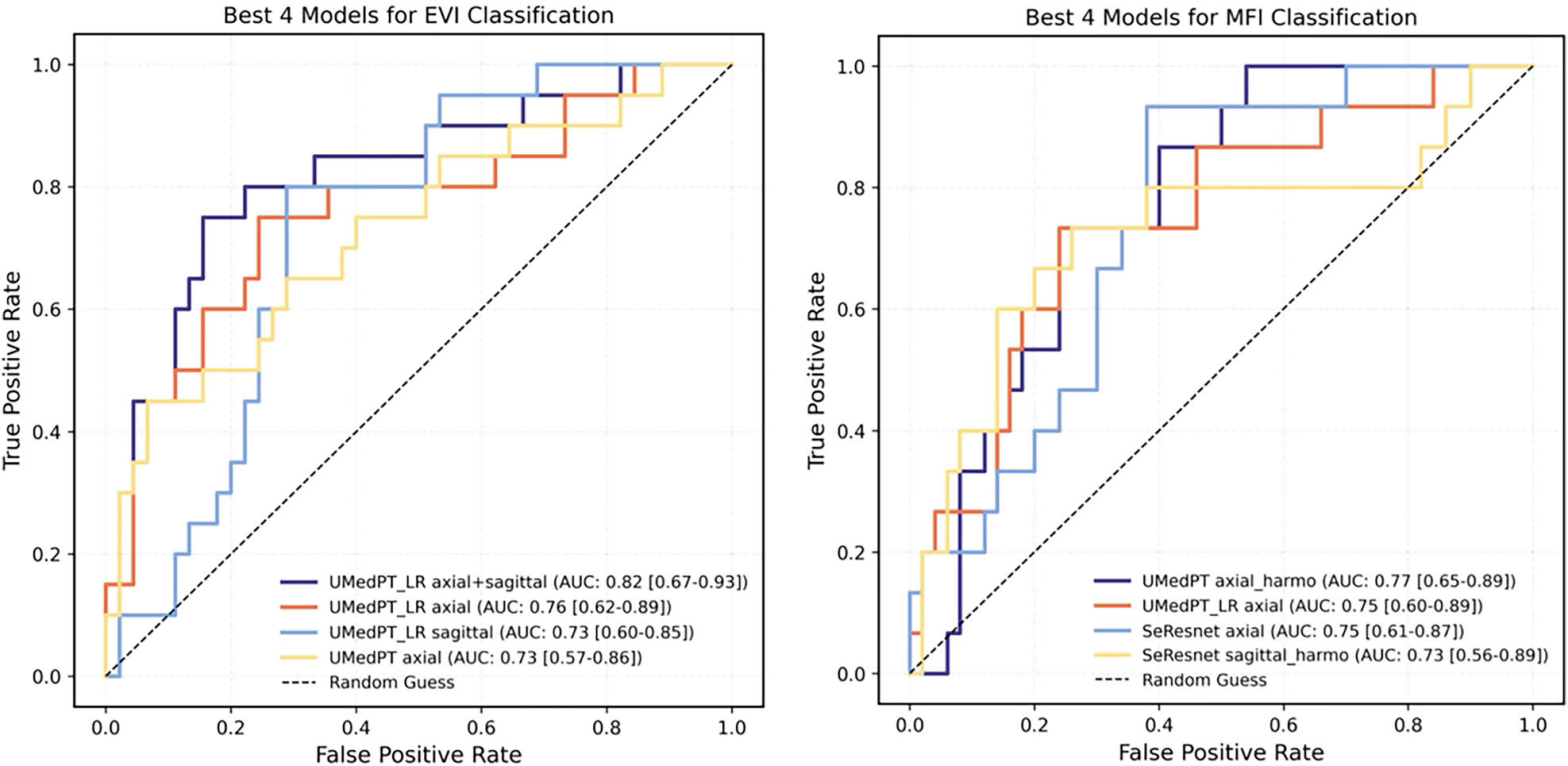
ROC curves of the top four models for classifying EVI (left) and MFI (right) on the CHAIMELEON’s test set. For EVI, UMedPT_LR with multiplanar fusion (axial + sagittal planes) achieved the highest AUC = 0.82. For MFI, the best performance was UMedPT trained on axial harmonized patches (AUC = 0.77). “Axial” refers to the original axial patch, and “axial_harmo” indicates the axial harmonized patch

For MFI classification, UMedPT demonstrated the best performance on axial harmonized patches, achieving an AUC of 0.77 and balanced accuracy of 0.72, representing the highest MFI result across all conditions (Table 4) and exceeding the CHAIMELEON challenge winner (AUC = 0.75). UMedPT_LR performed comparably on axial original patches (AUC 0.75, balanced accuracy 0.71; Table 4), while sagittal results were uniformly lower, with modest gains from SeResNet on harmonized sagittal images (AUC 0.73; Table 4). Multiplanar fusion of axial and sagittal patches using UMedPT_LR yielded an AUC of 0.71, which did not surpass the best single-plane performance. This pattern is also evident in the ROC curves (Fig. 4, right), where UMedPT on harmonized axial images achieves the best performance.

**Table 4:** Diagnostic performance of different models for classifying MFI on axial and sagittal T2-weighted MRI, before and after harmonization (test set)

Plane	Model	AUC (95% CI)	Sensitivity (95% CI)	Specificity (95% CI)	F1 (95% CI)	Balanced_acc (95% CI)
Axial original	SeResnet	0.75 (0.61–0.87)	0.73 (0.50–0.93)	0.64 (0.50–0.78)	0.50 (0.30–0.67)	0.69 (0.55–0.81)
	UMedPT	0.70 (0.55–0.84)	0.60 (0.36–0.83)	0.66 (0.53–0.78)	0.44 (0.24–0.62)	0.63 (0.50–0.78)
	UMedPT_LR	0.75 (0.60–0.89)	0.60 (0.33–0.85)	0.82 (0.71–0.93)	0.55 (0.31–0.74)	0.71 (0.57–0.85)
Axial harmonized	SeResnet	0.74 (0.58–0.87)	0.60 (0.35–0.84)	0.70 (0.57–0.82)	0.46 (0.26–0.65)	0.65 (0.52–0.79)
	UMedPT	0.77 (0.65–0.89)	0.73 (0.50–0.94)	0.70 (0.57–0.82)	0.54 (0.34–0.71)	0.72 (0.59–0.85)
	UMedPT_LR	0.72 (0.59–0.86)	0.40 (0.14–0.67)	0.90 (0.81–0.97)	0.46 (0.19– 0.68)	0.65 (0.52–0.79)
Sagittal original	SeResnet	0.41 (0.27–0.59)	0.87 (0.67–1.0)	0.14 (0.06–0.24)	0.37 (0.22–0.50)	0.50 (0.39–0.60)
	UMedPT	0.61 (0.45–0.78)	0.53 (0.26–0.79)	0.66 (0.53–0.80)	0.40 (0.19–0.59)	0.60 (0.45–0.74)
	UMedPT_LR	0.62 (0.46–0.78)	0.40 (0.14–0.67)	0.80 (0.68–0.91)	0.39 (0.16–0.59)	0.60 (0.47–0.74)
Sagittal harmonized	SeResnet	0.73 (0.56–0.89)	0.73 (0.50–0.95)	0.70 (0.57–0.83)	0.54 (0.33–0.71)	0.72 (0.58–0.85)
	UMedPT	0.59 (0.44–0.75)	0.53 (0.29–0.80)	0.62 (0.48–0.76)	0.38 (0.18–0.56)	0.58 (0.43–0.72)
	UMedPT_LR	0.60 (0.46–0.76)	0.27 (0.07–0.50)	0.76 (0.64–0.88)	0.26 (0.07–0.46)	0.51 (0.40–0.65)

### Grad-CAM visualization

**Fig. 5:**
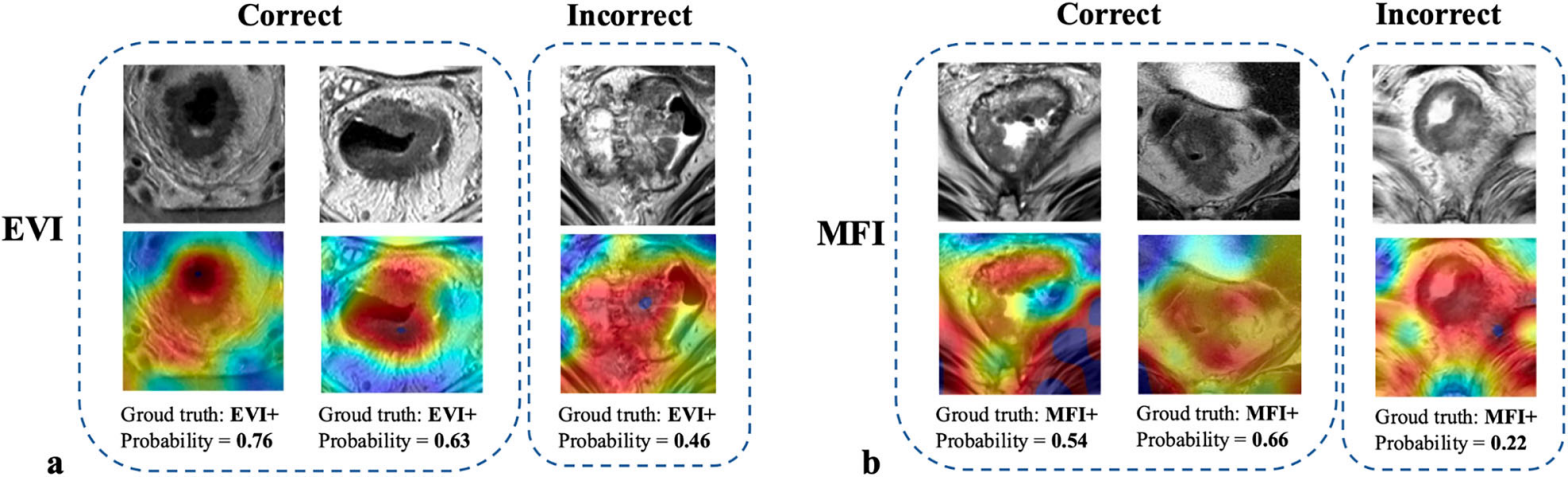
Grad-CAM visualizations for EVI and MFI classification. Examples of axial T2-weighted MR images (top row in each case) and corresponding Grad-CAM heatmaps (bottom row) are shown for EVI (**a**) and MFI (**b**). For each condition, two correctly classified cases (left) and one misclassified case (right) are presented. Grad-CAM heatmaps highlight image regions that contributed most to the model’s prediction. Ground truth labels and predicted probabilities are displayed beneath each case

To enhance interpretability, we applied Grad-CAM on axial T2-weighted MR images to highlight regions that most influenced the model’s predictions. As shown in Fig. 5, each pair displays the original image and its corresponding heatmap, highlighting areas of model attention.

For EVI classification, the model predominantly focused on localized areas near the tumor boundary. In contrast, MFI predictions consistently involved broader regions, particularly around the mesorectal fascia.

### RQS score

The proposed framework achieved an RQS 2.0 score of 25 out of 35 (71%), corresponding to Radiomics Readiness Level 6, which indicates a validated multi-center study demonstrating good reproducibility and potential for clinical translation. Detailed category-wise scores and figures are presented in the Appendix A4.

## Discussion

In this multi-center cohort, foundation-model pipelines (UMedPT/UMedPT_LR) outperformed conventional convolutional neural networks (CNNs) for both EVI and MFI classification tasks. For EVI, multiplanar UMedPT_LR achieved an AUC of 0.82 (vs SeResNet 0.52–0.57), indicating the value of pretrained representations and cross-plane feature aggregation. For MFI, UMedPT on axial harmonized patches reached 0.77. These findings position foundation models, multiplanar fusion, and harmonization as complementary strategies for robust rectal MRI analysis.

Previous radiomics-based studies have demonstrated promising results in rectal cancer classification. For EVI, Shu [33] et al and Liu [34] et al both reported improved AUC (≥ 0.83) when combining handcrafted radiomics features with clinical variables in integrated nomograms. For MFI, Liang [35] et al (*n* = 301) achieved an AUC of 0.91 using a combined model incorporating tumor and fascia region radiomics. However, these approaches typically depend on handcrafted feature engineering, which may hinder generalizability and scalability. A recent deep learning study by Cai [36] et al developed an automated pipeline combining nnU-Net segmentation with an MLNet classifier and achieved an external AUC of 0.76 for EVI classification using DWI across nine centers. Our foundation model–based framework achieved higher EVI performance (AUC = 0.82) despite being fine-tuned on a smaller three-center dataset (*n* = 331), highlighting the superior generalizability and data efficiency of foundation model representations over task-specific CNNs.

Most prior studies on rectal cancer MRI local staging have focused on axial T2-weighted images, with sagittal images mainly used for tumor localization or assessment of the anal canal and pelvic floor [7, 12]. To our knowledge, this is one of the first studies to quantitatively compare axial and sagittal T2-weighted images and multiplanar fusion for automated classification of EVI and MFI. In our experiments, the axial plane consistently yields higher performance for both tasks, likely due to its broader coverage of vascular and fascial structures. Multiplanar fusion improved performance when complementary information was present across planes [37], though gains were limited when one orientation dominated. This may highlight the importance of explicit plane selection in rectal MRI AI applications.

We also observed that harmonization improved MFI classification, particularly for UMedPT on axial images, but reduced performance for EVI. This suggests that excessive harmonization may attenuate subtle diagnostic cues [38, 39], especially those reflecting vascular abnormalities. These results indicate that harmonization may not be universally beneficial and should be adapted to the underlying biological question and the model’s feature dependencies [40].

This study has several limitations. First, although data were obtained from three centers, we did not perform a center-wise validation, and center-specific effects may therefore not be fully captured. Second, the overall sample size was modest (*n* = 331), including 66 cases in the test set, which may limit statistical power and generalizability. While the strong feature representation of foundation models may have mitigated some of these effects [41, 42], further validation in larger, multi-institutional cohorts, and independent external datasets are warranted. Third, while Grad-CAM was used to illustrate representative correct and incorrect predictions, no systematic error analysis was performed due to the limited size of the test cohort. Future work should also include prospective validation across broader populations, integration of diffusion-weighted and dynamic contrast-enhanced sequences, and longitudinal outcome prediction to support individual treatment decisions. Finally, in silico trials comparing diagnostic performance across radiologists alone, AI alone, and AI-assisted radiologists would provide valuable evidence of clinical translation, as demonstrated in our previous studies [43, 44].

## Conclusion

In summary, the Foundation models UMedPT and UMedPT_LR achieved state-of-the-art performance for EVI and MFI classification from multi-center rectal MRI. Multiplanar fusion (axial + sagittal planes), frequency domain harmonization, and consistent center-cropped rectal patches contributed to performance gains. Our findings highlight the potential of foundation models in rectal cancer staging and support their future application in clinical workflows.

## Supplementary information

ELECTRONIC SUPPLEMENTARY MATERIAL

## Data Availability

The multicentre MRI datasets used in this study are subject to institutional and EU data protection regulations and therefore cannot be publicly shared. An earlier version of this manuscript was made available as a preprint on arXiv (10.48550/arXiv.2505.18058). The preprint was subsequently updated during the review process.

## Abbreviations

AI: Artificial intelligence
AUC: Area under the curve
CI: Confidence intervals
CNNs: Convolutional neural networks
CRM: Circumferential resection margin
EVI: Extramural vascular invasion
Grad-CAM: Gradient-weighted class activation mapping
MFI: Mesorectal fascia invasion
MLP: Multilayer perceptron
nCRT: Neoadjuvant chemoradiotherapy
ROC: Receiver operating characteristic
RQS: Radiomics quality score
SeResNet: Squeeze and excitation residual network
UMedPT: Universal biomedical pretrained transformer
UMedPT_LR: Logistic-regression variant using frozen UMedPT features

## Code availability

The code will be made publicly available at https://github.com/yumengzhang97/foundation-model-rectal-mri upon publication.
