# Supplementary material for: A pre-trained foundation model framework for multiplanar MRI classification of extramural vascular invasion and mesorectal fascia invasion in rectal cancer
Source: Insights Imaging. 2026 May 22;17:141. doi: 10.1186/s13244-026-02296-3 (PMC13197564; doi:10.1186/s13244-026-02296-3)
Supplement: Supplementary file 1 — ELECTRONIC SUPPLEMENTARY MATERIAL [file 13244_2026_2296_MOESM1_ESM.pdf]

A Pre-trained Foundation Model Framework for Multiplanar MRI Classification of Extramural Vascular Invasion and Mesorectal Fascia Invasion in Rectal Cancer

ELECTRONIC SUPPLEMENTARY MATERIAL

A1. Frequency domain harmonization methods

This method is designed to reduce scanner and protocol induced intensity differences while preserving clinically relevant anatomical features essential for tumor staging and invasion assessment. A curated reference set of 17 high-quality rectal T2w MRI cases was selected based on consistent acquisition parameters (pulse sequence, TE, TR) and optimal image quality, as assessed by an expert radiologist. These reference scans were used to define the harmonization target space, representing the diversity of tissue-contrast distributions observed across sites. Synthetic contrast perturbations were applied to these reference images by modifying their frequency domain representations via Fast Fourier Transform (FFT). A circular mask centered in the frequency matrix was used to iteratively vary spatial frequency content, simulating a range of inter-site contrast conditions. Each altered image was then reconstructed into the spatial domain using inverse FFT, yielding 300 synthetic variants per 2D slice.

A self-supervised convolutional autoencoder was trained to reconstruct harmonized images from these contrast-altered inputs. The method is considered self-supervised because it synthetically perturbed images as inputs and the original reference images as targets, eliminating the need for manual labels. The architecture was based on a modified U-Net3+[1] with a VGG16[2] encoder pretrained on ImageNet, enhanced by learnable convolutional pooling and skip connections. Training was performed in two stages: (i) freezing the encoder while training decoder layers on 40,000 synthetic images followed by (ii) fine-tuning the entire model on additional 50,320 images for 200 epochs. The optimization used a composite loss combining mean squared error and L2 regularization to ensure fidelity in image reconstruction. This harmonization model produces standardized contrast outputs from heterogeneous MRI inputs and its outputs were included in our pipeline to improve robustness in downstream classification tasks.

A2. SeResNet baseline

We employed a modified version of Squeeze and Excitation Residual Network (SeResNet)-34 as the backbone network for classification, tailored to the anisotropic spatial resolution typical in pelvic MRI. To preserve spatial detail while minimizing feature loss through early downsampling, we customized the network stem to use a convolutional kernel of size 3×3×1 and applied anisotropic strides of (2,2,1) in the first two stages. LeakyReLU was used in place of ReLU to improve gradient flow in low-contrast regions.

The model comprises five sequential ResidualEncoder stages, with output channel sizes of 32, 64, 128, 256, and 512, respectively. The convolutional kernel size for the first two stages are 3×3×1 and 3×3×3 for the remaining ones. The stride configuration is (2,2,1) for the first two stages and the final stages, followed by (2,2,2) for the remaining stages. Each stage consists of 1, 3, 4, 6, and 3 residual blocks[3], respectively. All residual units are enhanced with squeeze-and-excitation (SE)[4] modules, enabling adaptive channel-wise feature recalibration. Following the final stage, a 3D global average pooling layer compresses the spatial dimensions, and the resulting feature vector is passed to a fully connected layer with 512 units. A final sigmoid-activated output layer produces binary predictions for EVI or MFI classification. The full architecture is summarized in **Figure A1.**

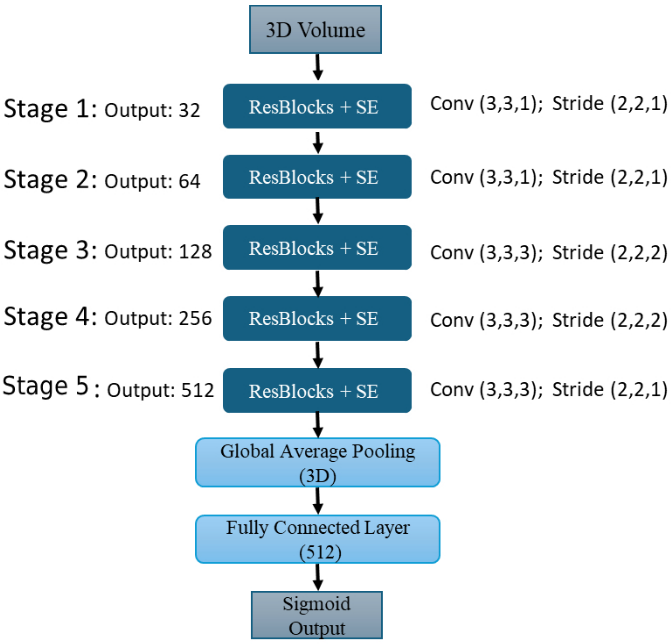

Figure A1. Architecture of the modified SeResNet used for EVI and MFI classification. The model consists of five convolutional stages, each comprising residual blocks enhanced with SE modules. Anisotropic convolution kernels and strides (e.g., 3×3×1 with stride 2×2×1) are used in the early stages to accommodate the non-isotropic resolution of pelvic MRI. The final feature map is globally pooled and passed through a 512-unit fully connected layer, followed by a sigmoid output for binary classification. Channel dimensions are progressively expanded across stages, as shown on the left.

A3. Data augmentation parameters

Table A1 The parameters of data augmentation(applied with MONAI)

Transform	Probability	Parameters
RandZoomd	0.2	min_zoom=0.9, max_zoom=1.1
RandAffined	0.5	rotate_range=rotate_range=(0, 0, π/15), shear_range=(0.1, 0.1, 0.1), scale_range=(0.1, 0.1, 0.1), padding_mode='border'
RandFlipd	0.2	spatial_axias=0
RandGaussianNoised	0.1	mean=0.0, std=0.1
RandGaussianSmoothd	0.1	sigma_x=(0.5, 1), sigma_y=(0.5, 1), sigma_z=(0.5, 1)
RandScaleIntensityd	0.2	factors=(0.8, 1.2)
RandAdjustContrastd	0.2	gamma=(0.8, 1.2)
RandBiasFieldd	0.1	coeff_range=(0.1, 0.2)
RandGibbsNoised	0.1	alpha=(0.6, 0.8)

A4. Radiomics Quality Score 2.0

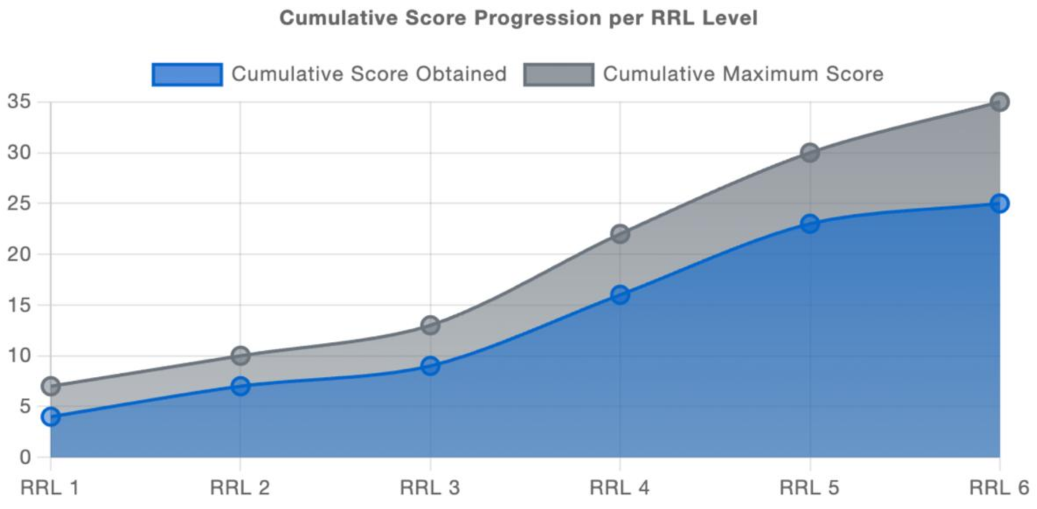

Figure A2. Evolution of score by RRL level. Cumulative score obtained (blue) versus cumulative maximum score (grey) across RRL1–RRL6.

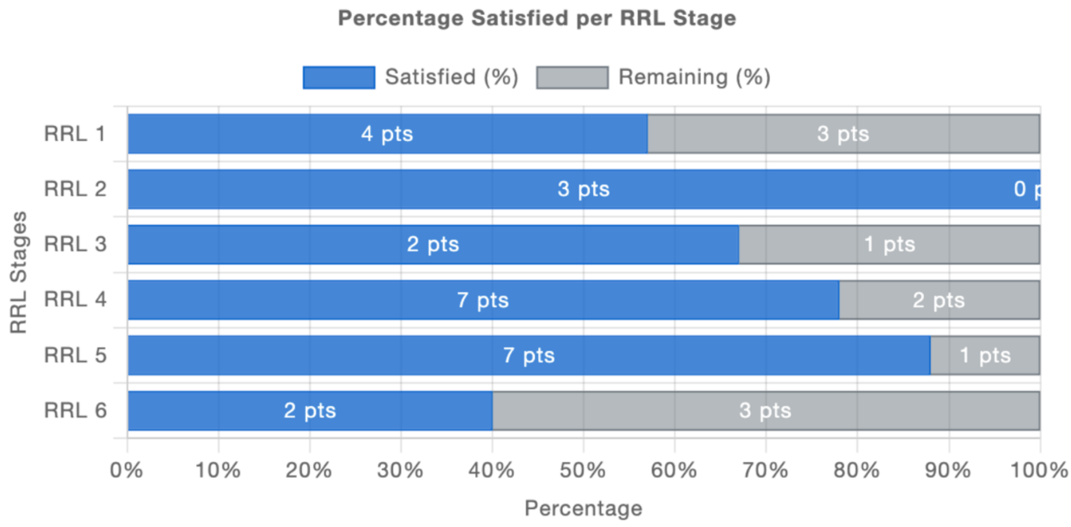

Figure A3. Points satisfied per stage. Stacked horizontal bars show, for each RRL stage, the percentage of points satisfied (blue) versus remaining (grey); in-bar labels indicate the number of satisfied points.

Table A2 Detailed Radiomics Quality Score (RQS 2.0) evaluation for the foundation model-driven framework for rectal cancer classification. The study achieved an RQS 2.0 score of 25/35 (Radiomics Readiness Level 6), reflecting strong methodological transparency, validation, and interpretability consistent with the RQS 2.0 framework

No.	Criteria	Selected Option	Points	Explanation
**RRL 1 - Foundational Exploration**				
1	Unmet Clinical Need – Unmet clinical need (UCN) defined.<br>● UCN is agreed upon and defined by more than one center.<br>● UCN is defined using an established consensus method such as the Delphi method.	Implemented: More than one center (+1)	1	The study addresses the unmet need for reliable MRI-based detection of EVI and MFI in rectal cancer, agreed upon by three centres within the EU CHAIMELEON project.
2	Hardware Description – Detailed description of the imaging hardware used, including model, manufacturer, and technical specifications.	Implemented (+1)	1	Different MRI scanner manufacturers used across centres (Siemens, Philips, GE) are detailed in the manuscript table to document hardware variability.
3	Image Protocol Quality – Five levels of image protocol quality for TRIAC:<br>● Level 0: Protocol not formally approved.<br>● Level 1: Approved with a reference number in the institutional archive.<br>● Level 2: Approved with formal quality assurance (recommended minimum for prospective trials).<br>● Level 3: Established internationally; published in guidelines and peer-reviewed papers.<br>● Level 4: Future proof (follows TRIAC Level 3, FAIR principles, retains raw data).	Not implemented	0	The study used retrospectively collected multi-center MRI data (CHAIMELEON project) acquired under heterogeneous clinical protocols without a unified TRIAC-compliant acquisition standard. Although no formal acquisition QA was performed, frequency domain harmonization was applied to mitigate scanner-related variability.
4	Inclusion and Exclusion Criteria – Detailed criteria for patient selection in studies, including rationale.	Implemented (+1)	1	Detailed inclusion/exclusion criteria were defined and are documented in the Methods (Dataset) section.
5	Diversity and Distribution – Identify potential biases before the project (demographics, socioeconomic, geographic, medical profiles).	Implemented (+1)	1	Patient demographics (age, gender) were summarised in Table 1. Data were collected from three hospitals in different regions, providing limited geographic and institutional diversity to reduce single-center bias.
**RRL 2 - Data Preparation**				
7	Preprocessing of Images – Apply steps to standardize images with clear reasoning.	Implemented (+1)	1	Preprocessing steps are fully described in the Methods (Preprocessing) section.
8	Harmonization – Use image-level (e.g. CycleGANs) or feature-level (e.g. ComBat)	Implemented (+1)	1	A self-supervised frequency domain harmonization strategy was applied to reduce scanner variability across centres.

	harmonization techniques.			
10	Automatic Segmentation – Use an automated segmentation algorithm for ROI definition.	Implemented (+1)	1	Rectum patch were automatically localised using the TotalSegmentator tool.
**RRL 3 - Prototype Model Development**				
13	HCR + DL Combination – Compare and explore the synergistic combination of handcrafted radiomics and deep learning models.	Not implemented	0	Only deep learning–based features from the UMedPT framework were used; handcrafted radiomics features were not combined or compared.
14	Multivariable Analysis – Incorporate non-radiomics features (clinical, genomic, proteomic) to yield a holistic model.	Implemented (+2)	2	Age was integrated with the UMedPT_LR multiplanar model (axial+sagittal) as a clinical covariate. No significant improvement was observed (EVI: AUC 0.73 vs 0.82; MFI: AUC 0.71 vs 0.71).
**RRL 4 - Internal Validation**				
15	Single Center Validation – Validation performed on data from the same institute without retraining or adapting the cut-off value.	Implemented (+1)	1	Data from three centers were combined and divided into training, validation, and test sets.
16	Cut-off Analyses – Identify optimal thresholds (e.g., using Youden's Index) for classification or survival analysis.	Implemented (+1)	1	A fixed probability threshold of 0.5 was used in line with CHAIMELEON challenge requirements.
17	Discrimination Statistics – Report discrimination metrics (e.g., ROC curve, sensitivity, specificity) with significance (p-values, CIs).<br>● Statistic reported<br>● With Resampling method	Resampling method applied (+2)	2	Discrimination metrics (ROC, AUC, sensitivity, specificity, 95% CI) were reported, and statistical significance assessed via resampling (e.g. bootstrapping).
18	Calibration Statistics – Report calibration metrics (e.g., calibration-in-the-large, slope, plots).	Implemented (+1)	1	Calibration plots are in the Supplementary materials.
19	Failure Mode Analysis – Document model limitations with examples of edge cases.	Implemented (+1)	1	Model limitations were discussed in the Discussion section, including reduced EVI performance with harmonisation and lower robustness of sagittal plane classification.
20	Open Science and Data – Make code and data publicly available.<br>● Open scans (+1)<br>● Open segmentations (+1)<br>● Open code (+1)	One aspect (+1)	1	The code will be publicly available via GitHub repository: https://github.com/yumengzhang97/foundation-model-rectal-mri
**RRL 5 - Capability Testing**				
21	Multi-center Validation – Validation with data from multiple institutes ensuring no overlap:<br>● One external institute<br>● Two or more external institutes<br>● Third-party platform with completely unseen data	Two or more institutes (+2)	2	Validation of the trained model was carried out on more than one centers.

22	Comparison with 'Current Clinical Standard' – Assess model agreement or superiority versus the current gold standard (e.g., TNM staging).	Implemented (+2)	2	Model performance was compared with the current clinical gold standard(histopathological assessment).
23	Comparison to Previous Work – Compare performance with published HCR signatures or DL algorithms.	Implemented (+1)	1	The proposed model was compared with previously the best performance of CHAIMELEON challenge.
24	Potential Clinical Utility – Report on the current and potential clinical application (e.g., decision curve analysis).	Implemented (+2)	2	The study discussed the model's potential clinical application in the discussion part.
**RRL 6 - Trustworthiness Assessment**				
25	Explainability – Apply explainability tools (e.g., SHAP for HCR, GradCAM for DL) to clarify model predictions.	Implemented (+1)	1	Explainability was addressed using Grad-CAM visualizations.
26	Explainability Evaluation – Conduct qualitative and quantitative evaluations of interpretability methods (e.g., checking consistency to adversarial perturbations).	Implemented (+1)	1	Qualitative explainability analysis was performed using Grad-CAM visualizations to identify image regions contributing most to model predictions. The highlighted areas were visually compared with anatomically relevant regions for EVI and MFI to assess interpretability consistency.
27	Biological Correlates – Detect and discuss biological correlates to deepen understanding of radiomics and underlying biology.	Not implemented	0	No molecular or histopathological correlation analyses were performed.
28	Fairness Evaluation and Mitigation – Evaluate model performance for biases and apply bias correction if needed.<br>● Fairness evaluated<br>● Bias correction applied	Not implemented	0	Fairness evaluation was not performed, as subgroup analyses (e.g., by center, gender, or age) were beyond the scope of this study. Future work will include bias assessment across demographic and institutional subgroups.
	**Total = 25/35 (71%)**			

A5. Calibration curves

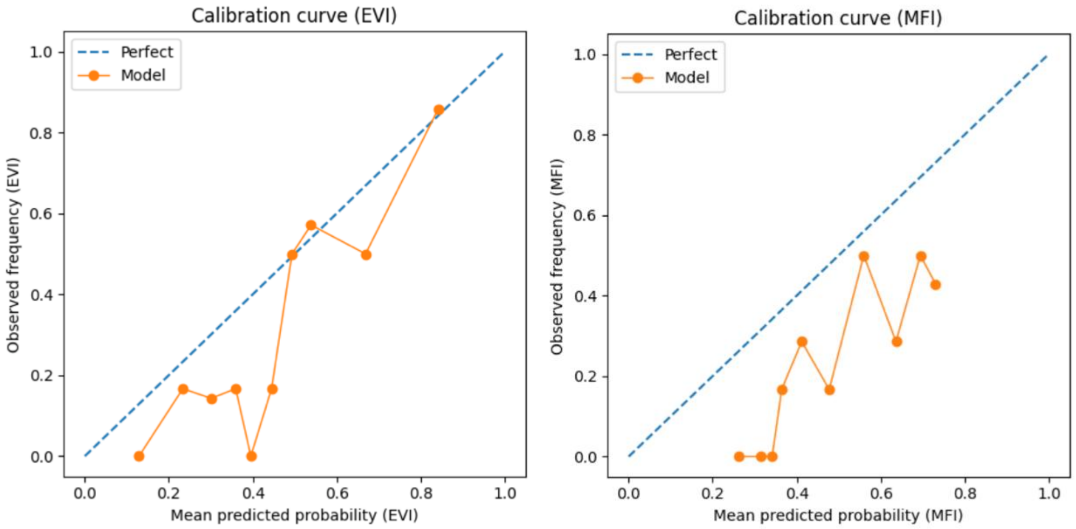

Figure A4. Calibration curves of the best EVI and MFI models on the test set (10 quantile bins). **EVI**—AUC = 0.82, Brier = 0.176, slope = 1.04, intercept = −0.69, ECE = 0.145; **MFI**—AUC = 0.77, Brier = 0.211, slope = 1.22, intercept = −1.28, ECE = 0.248.

A6. Relationship between radiological MFI and pathological CRM

Table A3 Cross-tabulation of radiological MFI and pathological CRM status

	**CRM +**	**CRM -**
**MFI +**	10 (15%)	55 (85%)
**MFI -**	10 (4%)	227 (96%)

Analysis was restricted to cases with available pathological CRM data (n =302). Cases with missing CRM status were excluded. MFI = mesorectal fascia invasion; CRM = circumferential resection margin; “+” indicates presence of the feature. Values are counts, with row-wise percentages in parentheses.

A7. Supplementary References

1. Huang H, Lin L, Tong R, et al (2020) UNet 3+: A full-scale connected UNet for medical image segmentation. arXiv [eess.IV]
2. Simonyan K, Zisserman A (2014) Very deep convolutional networks for large-scale image recognition. arXiv [cs.CV]
3. He K, Zhang X, Ren S, Sun J (2016) Deep residual learning for image recognition. In: 2016 IEEE Conference on Computer Vision and Pattern Recognition (CVPR). IEEE
4. Hu J, Shen L, Albanie S, et al (2017) Squeeze-and-Excitation Networks. arXiv [cs.CV]
